# Beauvericin Ameliorates Experimental Colitis by Inhibiting Activated T Cells via Downregulation of the PI3K/Akt Signaling Pathway

**DOI:** 10.1371/journal.pone.0083013

**Published:** 2013-12-10

**Authors:** Xue-Feng Wu, Rui Xu, Zi-Jun Ouyang, Cheng Qian, Yan Shen, Xu-Dong Wu, Yan-Hong Gu, Qiang Xu, Yang Sun

**Affiliations:** 1 State Key Laboratory of Pharmaceutical Biotechnology, School of Life Sciences, Nanjing University, Nanjing, Jiangsu Province, China; 2 Department of Clinical Oncology, the First Affiliated Hospital of Nanjing Medical University, Jiangsu Province, China; Duke University Medical Center, United States of America

## Abstract

Crohn's disease is a common, chronic inflammatory bowel condition characterized by remission and relapse. Accumulating evidence indicates that activated T cells play an important role in this disease. In the present study, we aimed to examine the effect of beauvericin, a natural cyclic peptide, on 2,4,6-trinitrobenzene sulfonic acid (TNBS)-induced colitis in mice, which mimics Crohn's disease. Beauvericin significantly reduced weight loss, diarrhea and mortality, accompanied with notable alleviation of macroscopic and microscopic signs. In addition, this compound decreased serum levels of tumor necrosis factor (TNF)-α and interferon (IFN)- γ in a concentration-dependent manner in mice with experimental colitis. These effects of beauvericin are attributed to its inhibition on activated T cells. Flow cytometry and immunoblot assay data showed that beauvericin suppressed T-cell proliferation, activation and IFN-γ-STAT1-T-bet signaling and subsequently led to apoptosis of activated T cells by suppressing Bcl-2 and phosphorylated Bad as well as increasing cleavage of caspase-3, -9, -12 and PARP. Furthermore, inhibition of PI3K/Akt signaling, which was an upstream regulator of cell activation and survival in activated T cells, contributed to the effect of beauvericin. Overall, these results supported beauvericin as a novel drug candidate for the treatment of colonic inflammation mainly by targeting PI3K/Akt in activated T cells.

## Introduction

Crohn's disease is a chronic inflammatory bowel condition, characterized by remission and relapse, with a high incidence of 27–48 cases per 100,000 persons per year in western countries [1]. Although the etiology of the disease is uncertain, it has been suggested that a key role may be the mucosal immune system activating in response to bacterial antigens, with consequent pathological cytokine production [2]. Moreover, the mucosa of patients with established Crohn's disease are dominated by CD4^+^ T lymphocytes, which are distinguished by their capacity for producing interferon-γ (IFN-γ) and interleukin-2 (IL-2) [3], [4], [5]. To mimic this disease in mice, a chemically induced model of colonic inflammation has been developed. Intrarectal delivery of 2,4,6-trinitrobenzene sulfonic acid (TNBS) causes transmural inflammation, along with weight loss and histopathological features similar to Crohn's disease.

Medications used to treat Crohn's disease symptoms include corticosteroids, antibiotics, and immunomodulators, such as cyclosporine A, methotrexate, and the TNF-α monoclonal antibody infliximab [6], [7]. However, prolonged use of these medications can cause significant side-effects. Therefore, there is an urgent need for potent new agents that target signaling in activated T lymphocytes. The serine/threonine kinase Akt (protein kinase B) is activated upon T-cell antigen receptor (TCR) engagement or upon activated phosphatidylinositide (PI) 3-kinase expression in T lymphocytes [8]. Previous studies (our own and others) have demonstrated that Akt influences T cell activation and survival by inhibiting apoptotic processes [9], [10]. Akt signaling also regulates Th1 differentiation [11], [12]. It has been demonstrated that murine models featuring an activated PI3K/Akt/mTOR signaling pathway in lymphocytes exhibit signs of systemic autoimmunity [13] that link this pathway to autoimmune disorders. Thus, inhibiting PI3K/Akt signaling may prevent inflammatory bowel disease development mediated by activated T lymphocytes.

While screening a variety of compounds, we discovered that beauvericin, a cyclic hexadepsipeptide, showed potent immunomodulatory effects on PI3K/Akt phosphorylation during T-cell activation. Chemically, beauvericin is a cyclic hexadepsipeptide with alternating N-methyl-L-phenylalanine and hydroxyisovaleric acid residues. Although beauvericin was synthesized in 1971 [14], [15], there have been few reports of its biological activity other than antibacterial and anticancer activity [16], [17]. In the present study, beauvericin was found to ameliorate inflammatory bowel disease in mice and its mechanisms were related to inhibiting activated T lymphocytes via downregulation of PI3K/Akt signaling.

## Materials and Methods

### Ethics Statement

All procedures were strictly performed in accordance with the Guide for the Care and Use of Laboratory Animals (The Ministry of Science and Technology of China, 2006). All animal experiments were approved by Nanjing University Animal Care and Use Committee, and were designed to minimize suffering and the number of animals used.

### Animals

Specific pathogen-free female BALB/c mice (aged 8–12 weeks, weight 18–22 g) were obtained from the Yangzhou University Animal Center (Yangzhou, China) and housed in groups in an SPF facility under controlled temperatures (22±2°C) and photoperiods (12:12-h light:dark cycle). Mice were acclimated to these conditions for at least 2 days before use in experiments. For each group of experiments, mice were matched by age and body weight.

### Drugs and reagents

The following drugs and reagents were used. Beauvericin, cyclo(D-alpha-Hydroxyisovaleryl-L-N-methyl-Phe)3, was purchased from Sigma-Aldrich (St. Louis, MO, U.S.A.). RPMI-1640, FBS and CFSE Cell Proliferation Kit were purchased from Invitrogen (Carlsbad, CA). Antibodies against STAT1, phosphorylated STAT1 (Tyr 701), Akt, phosphorylated Akt (Thr 308), and phosphorylated Akt (Ser 473) were purchased from Cell Signaling Technology (Beverly, MA). Anti-cleaved Caspase-3, Caspase-9, Caspase-12, PARP, T-bet, Tubulin, Bad, Bcl-2 and anti-Actin were purchased from Santa Cruz Biotechnology (Santa Cruz, CA). Purified murine anti-CD3 (145-2C11) and purified anti-CD28 (37.51) were purchased from BD PharMingen (San Diego, CA). ELISA kits for murine IL-2, TNF-α, IFN-γ, IL-1β and IL-12 were purchased from Dakewe Biotech Co. Ltd (Shenzhen, China). Dexamethasone, 2,4,6-trinitrobenzenesulfonic acid (TNBS), concanavalin A (Con A), LY294002, 4′,6-diamidino-2-phenylindole (DAPI) and VO-OHpic were purchased from Sigma-Aldrich (St. Louis, MO). Recombinant murine IFN-γ was purchased from Peprotech (Rocky Hill, NJ). Annexin V-FITC (fluorescein isothiocyanate)/PI (propidium iodide) kit was purchased from BD Biosciences (San Jose, CA). 5,5′,6,6′-tetrachloro-1,1′,3,3′-tetraethyl-benzimidazolylcarbocyanine iodide (JC-1) was purchased from Molecular Probes (Eugene, OR). FITC-anti-CD4 was purchased from eBioscience (San Diego, CA). MACS Separation columns were purchased from Miltenyi Biotech (Bisley, UK). All other chemicals were purchased from Sigma-Aldrich (St. Louis, MO).

### Induction and evaluation of colitis

Mice were sensitized by painting 100 µL of 1% TNBS in ethanol onto the shaved skin of their abdomens 7 days before challenge. On the day of challenge, the mice were fasted for 20 h before the injection. A 3.5 F catheter was then carefully inserted through the anus into the colon; the tip was placed 4 cm from the anus. They were given 100 µL of 0.5% TNBS (in 50% ethanol to break the intestinal epithelial barrier) slowly injected into the lumen of the colon via a catheter fitted to a 1 mL syringe. In the sham control, mice received 50% ethanol only. The animals were kept vertical for 30 s, allowing TNBS and/or ethanol to permeate the entire colon, including the caecum and appendix, and then returned to their cages. Beauvericin (1, 2 or 4 mg/kg) and dexamethasone (1 mg/kg) were administered intraperitoneally once daily from day 0 to day 7. TNBS control mice received PBS alone. The animals were monitored once daily for weight, water, food consumption, morbidity, stool consistency, and the presence of blood in the feces and at the anus. The established and validated diarrhea index was calculated by assigning scores, as previously described [18]. Briefly, the following values were assigned: 0 =  normal, 2 =  loose stools, 4 =  watery diarrhea. After the experiment, the animals were sacrificed and rapidly dissected; the entire colon was quickly removed, and macroscopic scores were determined (blinded) on a scale from 0 to 9 based on criteria for inflammation: 1 Erythema; 2 Hemorrhage; 3 Edema; 4 Stricture formation; 5 Ulceration; 6 Fecal blood; 7 Presence of mucus; 8 Diarrhea; 9 Adhesions. Each parameter was awarded 1 point if observed after tissue examination [19]. Two days after TNBS challenge, segments of the colon were taken for histopathological essay and fixed in 10% normal buffered formalin, embedded in paraffin, sectioned to 5 µm thickness with a paraffin microtome, and mounted on microscope slides. Sections were stained with hematoxylin and eosin (H&E), and a histological score was calculated, evaluating damage to the epithelial mucosa as well as inflammatory infiltration. Histological grading was performed as previously described [20]. A maximum score of 8 indicated severe colitis, with a diffuse pattern of chronic changes.

### Analysis of cytokine profiles

Colons were obtained from mice and homogenized with lysis buffer to extract total protein on day 2 after TNBS challenge. Levels of cytokines TNF-α, IFN-γ, IL-1β and IL-12 were determined using ELISA kits following the manufacturer's instructions. For *in vitro* assays, the CD3^+^ T cells obtained from lymph nodes were treated with various concentrations of beauvericin in the presence of Con A (5 µg/mL) for 24 h; then the levels of cytokines TNF-α, IFN-γ, and IL-2 were determined. The threshold of detection was 10 pg/mL. The standard curve ranged from 0 to 2000 pg/mL.

### Measurement of cell proliferation

CD3^+^ T cells were isolated from lymph nodes and cell proliferation was evaluated with a modified CFSE assay. Cells were incubated with 5 µM CFSE at 37°C for 10 min and then quenched with ice-cold culture medium for 5 min. Cells were then washed and incubated in 24-well plates at a density of 5×10^6^/mL in RPMI 1640 medium (1.2 mL/well). The cells were stimulated with 5 µg/mL of Con A and cultured with various beauvericin concentrations for 72 h.

### Measurement of T cell activation

The CD3^+^ T cells obtained from lymph nodes were treated with various concentrations of beauvericin in the presence of Con A (5 µg/mL) for 12 h. The cells were harvested and washed twice with cold phosphate-buffered saline (PBS) and then incubated with anti-CD69 or anti-CD25 antibodies (FITC conjugated) for 30 min over ice before the flow cytometric analysis. Data were analyzed using Cell Quest software.

### Cell apoptosis

Cell apoptosis was determined by Annexin V/PI staining. The cells were measured by flow cytometry after addition of FITC-conjugated Annexin V and PI, as previously described [21]. Annexin V^+^ cells were considered apoptotic.

### Cell mitochondrial membrane potential assay

Cells were seeded in 12-well plates at a density of 1×10^6^ cells/well in RPMI 1640 medium, and treated with various concentrations of beauvericin for 20 h in the presence of Con A (5 µg/mL). The disruption of mitochondrial membrane potential was measured using JC-1 staining (10 µg/mL) by flow cytometry as previously reported.

### Western blot analysis

Western blot analyses were performed as previously described [22]. Briefly, cells were collected and treated with lysis buffer containing a protease inhibitor cocktail (Pierce). In some experiments, cells were separated into cytosolic and mitochondrial fractions using the ProteoExtract Cytosol/Mitochondria Fractionation Kit (Merck Bioscience, Bad Soden, Germany) according to the procedures provided by the manufacturer. Then proteins were fractionated by SDS-PAGE and electro-transferred to PVDF membranes. Various antibodies were used for blotting; the software Quantity One-4.6.5 (Bio-Rad Laboratories, Hercules, CA) was used for densitometric analysis.

### Immunohistochemistry

CD4^+^ T cell infiltration analysis was performed on paraffin-embedded colonic tissue sections (5 µm). The sections were deparaffinized, rehydrated and washed in 1% PBS-Tween. Then they were treated with 2% hydrogen peroxide, blocked with 3% goat serum and incubated for 2 h at room temperature with anti-CD4 FITC (1∶100). The slides were then counter-stained with DAPI for 2 min. The reaction was stopped by thorough washing in water for 20 min. Images were acquired by confocal laser-scanning microscope (Olympus FV1000). Images were randomly coded and transferred to a computer for further analysis.

### Statistical analysis

Results are expressed as mean ± SEM of three independent experiments in triplicate. Data were evaluated by a one-way analysis of variance (ANOVA) followed by Dunnett's test. The level of significance was set at a *P* value of 0.05.

## Results

### Beauvericin attenuated TNBS-induced colitis

**Figure 1:**
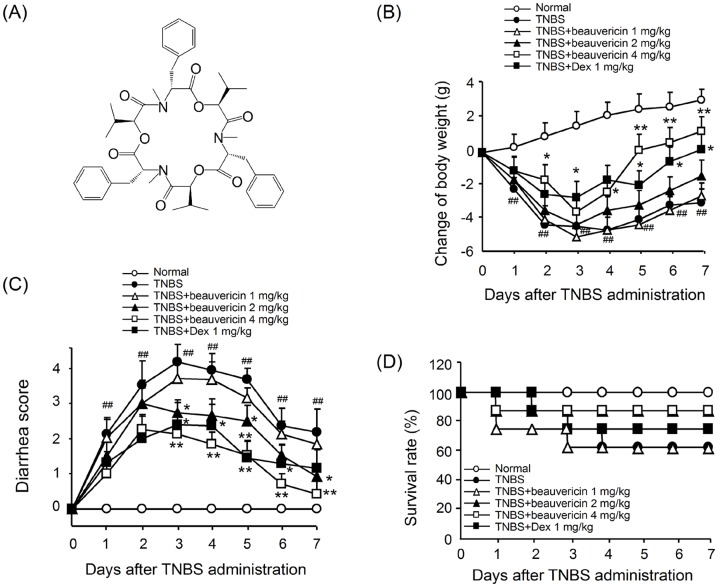
Improvement of TNBS-induced colitis in mice by beauvericin. (A) Chemical structure of beauvericin. (B) Body weight changes two days after TNBS challenge. (C) Diarrhea evaluations two days after TNBS challenge. (D) Survival rate during the colitis process. Data indicate mean ± SEM (n = 10). *P<0.05, **P<0.01, *vs.* TNBS control; ^#^ *P*<0.05, ^##^ *P*<0.01, *vs.* sham.

The structure of cyclic hexapeptide beauvericin is presented in Fig. 1A. To determine the immunomodulatory activity of beauvericin, we investigated its therapeutic efficacy in TNBS-induced T cell-mediated murine colitis. The intracolonic administration of TNBS in mice induced a severe illness that was characterized by body weight loss, stool consistency alterations, and bleeding, which peaked at day 2–3 after challenge, with mortality thereafter. Compared with the vehicle-treated group, beauvericin at 4 mg/kg dose restored body weight, as did dexamethasone (Fig. 1 B). Disease progression symptoms characterized by the appearance of significant diarrhea/loose feces with visible fecal blood were markedly improved by 4 mg/kg of beauvericin or 1 mg/kg of dexamethasone (Fig. 1 C). Survival was also increased by beauvericin (Fig. 1 D).

### Beauvericin inhibited pathological changes in TNBS-induced colitis

**Figure 2:**
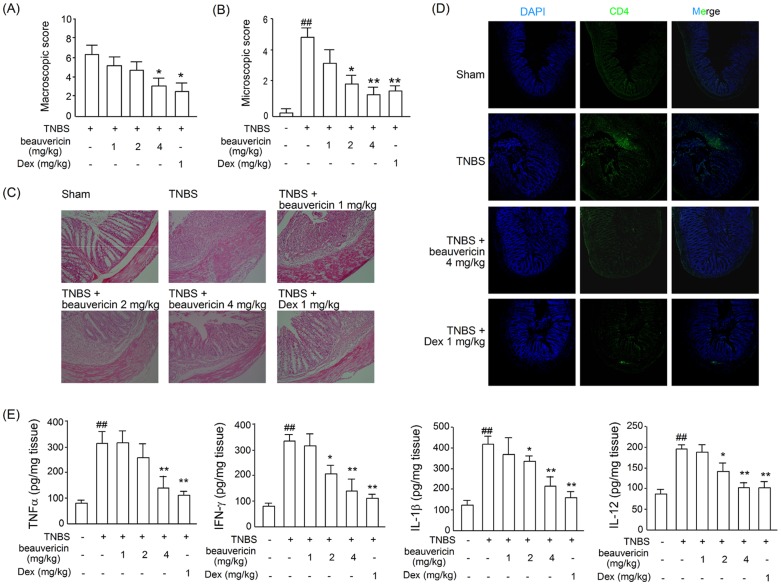
Effects of beauvericin on the appearances and CD4^+^ T cell infiltration of inflamed mouse colon. Colons were removed when mice sacrificed. (A) Macroscopic evaluation of colons after TNBS-induced colitis. (B) Microscopic scores for colons after TNBS-induced colitis. (C) Histopathological changes in colons. (D) Sections of colonic tissue were immunostained with DAPI (blue) and anti-CD4-FITC (green) and observed by confocal laser-scanning microscope. Data indicate mean ± SEM (n = 8). (E) The colonic levels of TNF-α, IFN-γ, IL-1β and IL-12 from mice with TNBS-induced colitis were determined by ELISA. *P<0.05, **P<0.01, *vs.* TNBS control; ^##^ *P*<0.01, *vs.* sham. Histopathological sections were stained with H&E. Amplification is 200× in (C) and 100× in (D).

A macroscopic evaluation of the distal colon and rectum after mice sacrifice revealed mucosal edema and hemorrhagic ulcerations. The most severely damaged colonic walls thickened from edema. Watery feces were observed in the control group. By comparison, normal stools were found in the colons of the drug-treated mice, which was indicative of a significant resolution of colitis (Fig. 2 A). At the same time, a histological analysis showed a loss of architecture and transmural immune cell infiltration extending through the mucosa and submucosa in the colon specimens from the TNBS-treated mice, while marked reductions in inflammatory response and mucosal ulcerations were observed after the beauvericin (4 mg/kg) or dexamethasone (1 mg/kg) treatment (Fig. 2 B–C).

### Beauvericin suppressed T cell infiltration into colonic tissue

To further investigate the mechanism of protection from colitis by beauvericin, we examined T cell infiltration in the colon tissues from each group. We observed a large number of CD4^+^ T cells in colonic samples from vehicle-treated TNBS mice. These infiltrated T cells were mainly located in the mucosa of the lesion site. In contrast, few infiltrating cells were detected in either beauvericin-treated or dexamethasone-treated colonic samples (Fig. 2 D). The suppressive effect of beauvericin correlated with its inhibition on serum levels of IFN-γ and TNF-α. Taken together, these results indicate that beauvericin reduces T cell activity, thereby ameliorating TNBS-induced colitis.

### Beauvericin regulated the profiles of cytokines in colons of mice with TNBS-induced colitis

Furthermore, to analyze the inflammatory mediators involved in colonic inflammation during TNBS-induced colitis, colon specimens from each group were picked out and homogenized for total protein extraction on day 2 after the TNBS challenge. Cytokine production was examined using ELISA. Our results demonstrated that the tissue levels of the cytokines IFN-γ, TNF-α, IL-1β and IL-12 were significantly increased after the TNBS administration, which suggested some role in intestinal inflammation. Beauvericin or dexamethasone treatment significantly reduced the levels of these inflammatory cytokines (Fig. 2 E). Cytokine level changes correlated with suppressed inflammation and colitis resolution.

### Beauvericin significantly inhibited T cell activation and proliferation

**Figure 3:**
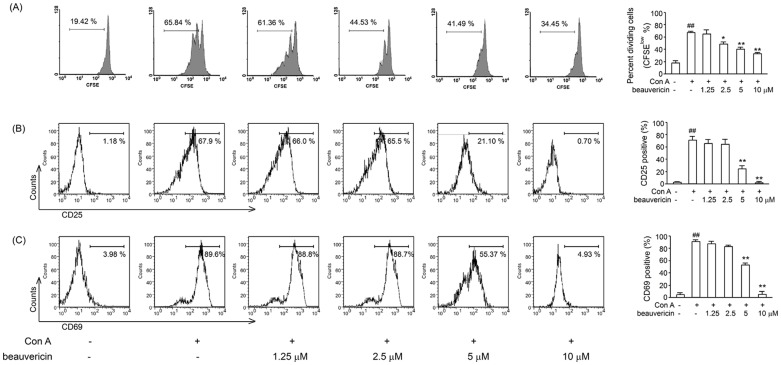
Effects of beauvericin on T lymphocyte proliferation and activation. (A) Beauvericin suppressed T cells proliferation. CD3^+^ T cells pretreated with CFSE (5 µM, 10 min) were incubated for 72 h at 37°C under 5% CO_2_ in the presence of Con A, with or without 1.25–10 µM beauvericin. Cell proliferation was measured by flow cytometry. (B–C) Beauvericin inhibited T cells activation. CD3^+^ T cells were stimulated in the presence of Con A (5 µg/mL) for 12 h, then CD69 and CD25 expression were determined by flow cytometry. Results represent mean ± SEM of three independent experiments. *P<0.05, **P<0.01, *vs.* control; ^##^ *P*<0.01, *vs.* sham.

The ameliorating effect of beauvericin in colitis prompted us to investigate potential regulatory mechanisms, such as how it affected lamina propria T-cell responses. We hoped to identify the signaling pathway through which beauvericin might regulate inflammatory diseases. To this end, CD3^+^ T lymphocytes were isolated from lymph node cells and tested *in vitro* for activation and proliferation in response to challenge. As shown in Fig. 3A, beauvericin dose-dependently inhibited the proliferation of T cells stimulated by Con A, a classic cell-activating mitogen. Furthermore, CD69 and CD25, activation markers in T cells, were significantly inhibited by beauvericin after Con A stimulation (5 µg/mL) for 12 h (Fig. 3B–C).

### Beauvericin inhibited IFN-γ/STAT1/T-bet signaling

**Figure 4:**
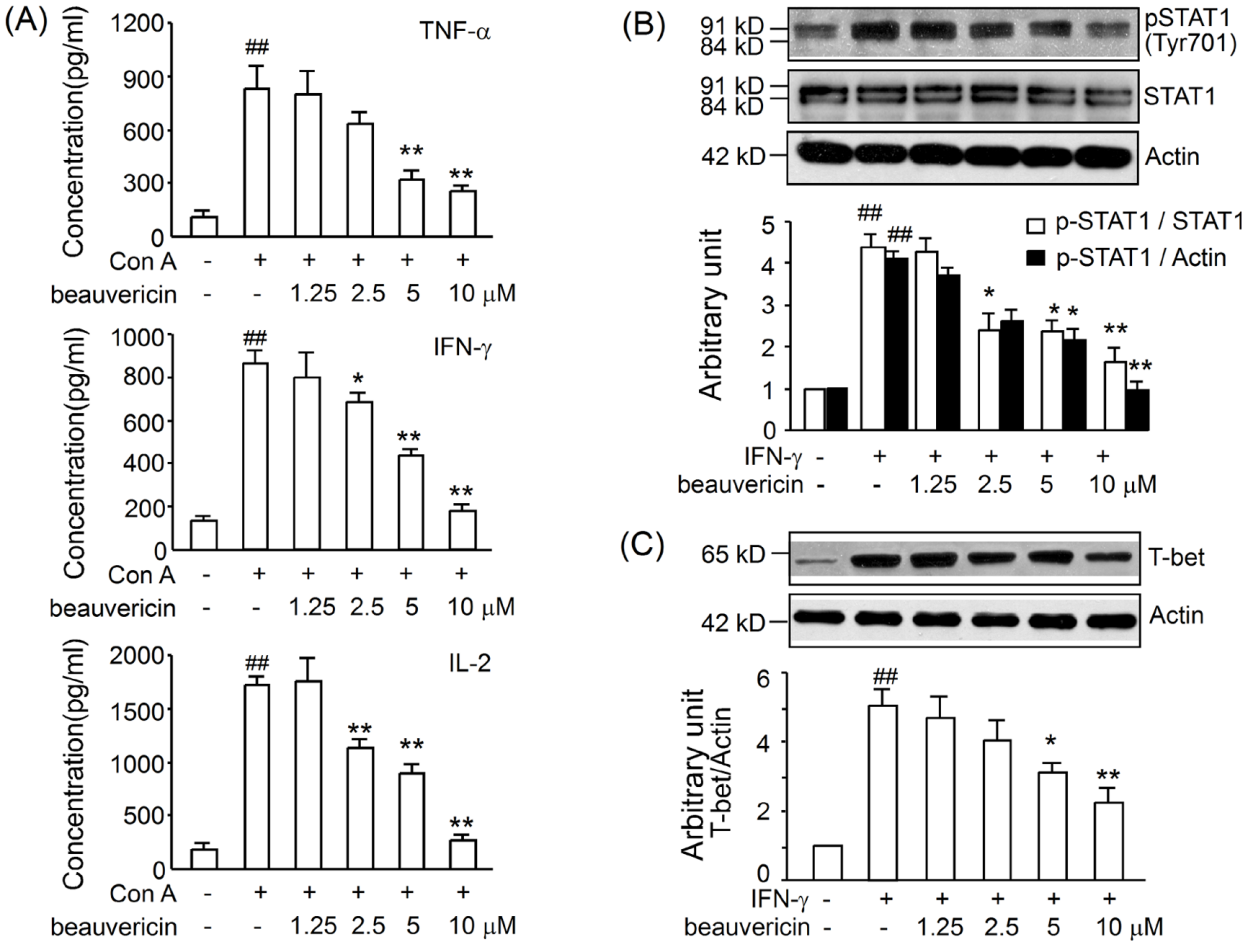
Effects of beauvericin on cytokine production, STAT1 phosphorylation, and T-bet expression in T lymphocytes. CD3^+^ T cells purified from lymph nodes (5×10^5^) were incubated with different concentrations of beauvericin in the presence of Con A for 24 h. The levels of TNF-α, IFN-γ and IL-2 (A) in supernatants of T cells were determined by ELISA (B, C) T cells were cultured with 2.5-10 µM beauvericin for 3 h, then treated with 25 ng/mL murine IFN-γ for 30 min (detection of pSTAT1, B) or 6 h (detection of T-bet, C). After the incubation, proteins were extracted and assessed by western blotting. The values are shown as mean ± SEM from three independent experiments. *P<0.05, **P<0.01, *vs.* control; ^##^ *P*<0.01, *vs.* sham.

To determine if beauvericin affects T cell cytokines *in vitro* as it does *in vivo*, we examined cytokine profiles and expression of phosphorylated STAT1 and T-bet in activated mouse T cells. CD3^+^ T cells were isolated and challenged with Con A (5 µg/mL) *in vitro*. In culture supernatants, cytokines commonly associated with T cell activation including IL-2, TNF-α, and IFN-γ were significantly downregulated (Fig. 4A). IFN-γ/STAT1/T-bet signaling is considered essential for T cell activation and cytokine production. Incubation of lymph node CD4^+^ T cells for 30 min with IFN-γ resulted in a marked enhancement of STAT1 tyrosine phosphorylation. Coincubation of IFN-γ-treated T cells with beauvericin (10 µM) completely inhibited phosphorylation of Tyr701 in STAT1 (Fig. 4B). A downstream molecule of STAT1, Th1-specific transcription factor T-bet, was also suppressed by beauvericin in a concentration-dependent manner after incubated with IFN-γ for 6 h *in vitro* (Fig. 4 C).

### Beauvericin induced apoptosis of activated T lymphocytes

**Figure 5:**
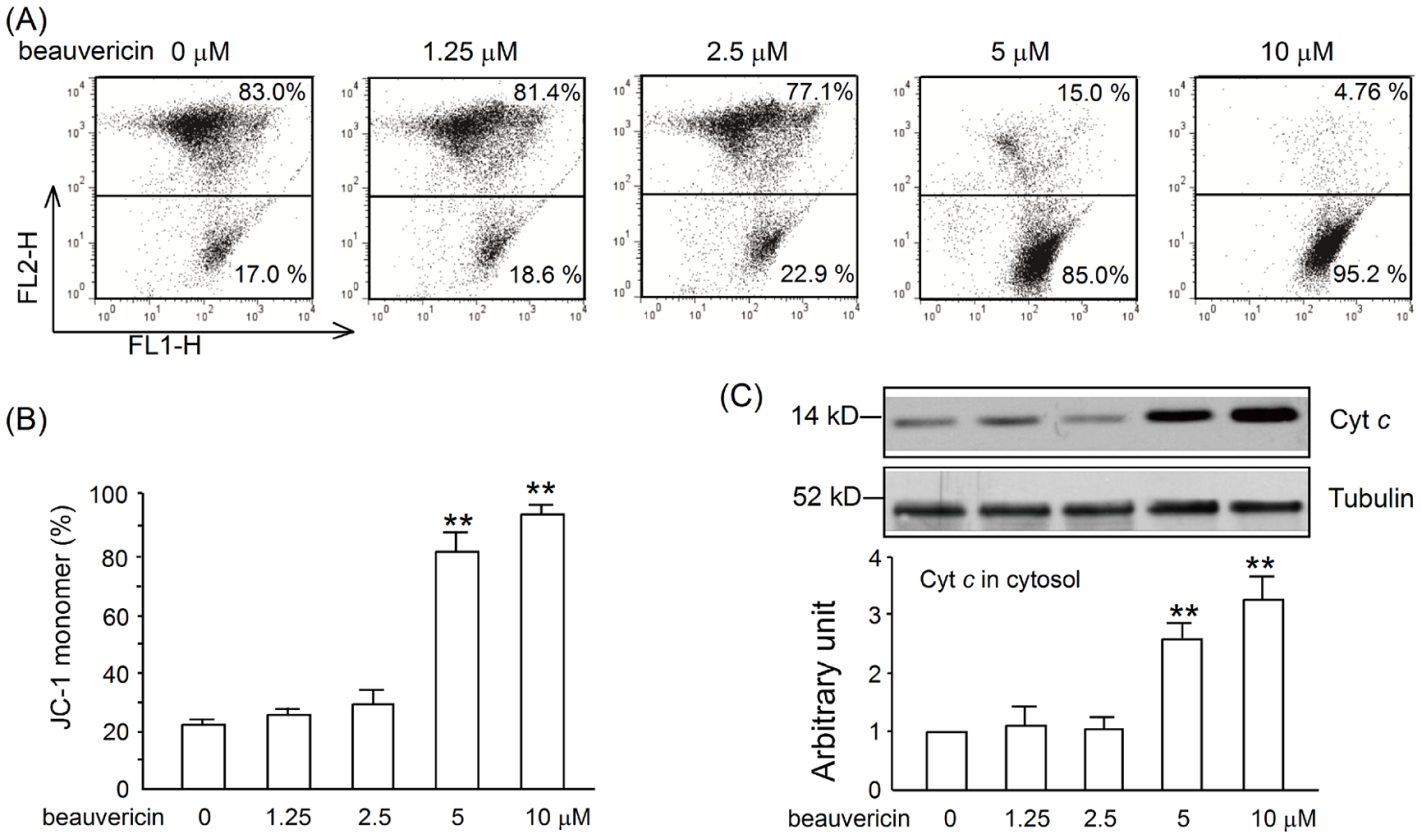
Effects of beauvericin on the collapse of mitochondrial membrane potential and release of cytochrome *c* in activated T cells. (A) Beauvericin induced the collapse of mitochondrial membrane potential. Purified CD3^+^ T cells from lymph nodes of BALB/c mice were treated with beauvericin for 20 h in the presence of 5 µg/mL Con A. Cells were harvested, and the disruption of mitochondrial membrane potential was measured using JC-1 staining (5 µg/mL, 10 min) by flow cytometry. Dots in the upper side indicate cells without mitochondrial disruption (JC-1 red aggregate). Dots in the lower side indicate cells with collapsed mitochondria membrane potential (JC-1 green monomers). (B) Mitochondrial membrane potential assay. (C) Cells were harvested and separated into cytosolic and mitochondrial fractions. Expression of cytochrome *c* in cytosol was analyzed by western blot analysis. Data represent mean ± SEM of 3 independent experiments. *P<0.05, **P<0.01 *vs.* drug untreated group.

**Figure 6:**
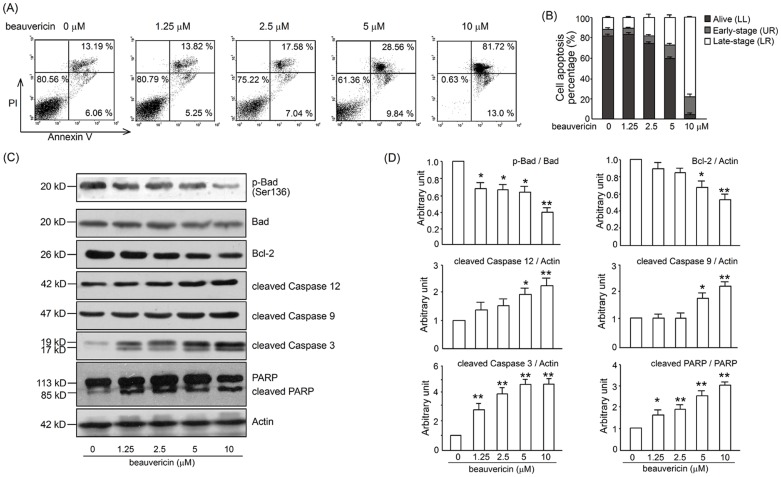
Effects of beauvericin on caspase-dependent apoptosis in Con A-activated T cells. Cells were seeded in 6-well plates and incubated with 1.25–10 µM beauvericin for 24 h in the presence of 5 µg/mL ConA. (A) Apoptosis was determined by Annexin V/PI staining. (B) Annexin V^+^/PI^−^ and Annexin V^+^/PI^+^ cells from 3 independent experiments are shown in columns. Representative western blot bands (C) and data summary (D) for cleaved caspase 3, 9, 12, PARP and Bcl-2/Bad in activated T cells after treatment with 1.25–10 µM beauvericin for 24 h. Data are expressed as a histogram of mean ± SEM of 3 independent experiments. *P<0.05, **P<0.01 *vs.* drug untreated group.

To examine the relationship between inhibition of proliferation and apoptosis, we incubated T cells with beauvericin in the presence of Con A. Collapse of mitochondrial membrane potential is a landmark event in apoptosis. Mitochondrial membrane potential was detected using JC-1 staining. We observed an obvious reduction in mitochondrial membrane potential in beauvericin-treated cells after Con A stilmulation for 20 h (Fig. 5A and B). We also examined mitochondrial and cytosolic proteins isolated from T cells treated with beauvericin relative to control. As shown in Fig. 5C, the compound greatly increased cytosolic cytochrome *c*. Moreover, T cell apoptosis was also detected using an Annexin V/propidium iodide staining assay 24 h after Con A administration. T cells activated with Con A and exposed to beauvericin underwent apoptosis in a dose-dependent manner (Fig. 6 A–B). In addition, expression of several apoptosis-related proteins was determined by western blot analysis. After being incubated with various concentrations of beauvericin (0 µM, 1.25 µM, 2.5 µM, 5 µM, or 10 µM) in the presence of Con A (5 µg/mL) for 24 h, Bcl-2 (the anti-apoptotic protein) was significantly downregulated, while its binding protein (Bad) was dephosphorylated. Caspases 3, 9, 12, and PARP were found to be cleaved in a dose-dependent manner (Fig. 6 C–D). These results indicated that beauvericin triggered apoptosis through a mitochondria-dependent pathway.

### Beauvericin inhibited PI3K/Akt signaling

**Figure 7:**
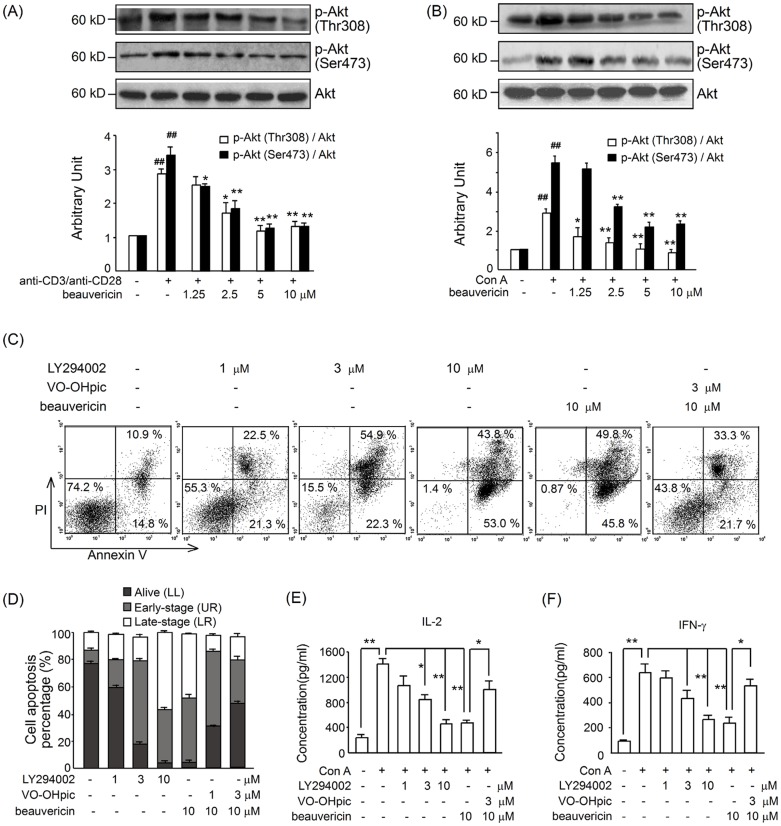
Effects of beauvericin on phosphorylated Akt in T cells activated by Con A or anti-CD3/anti-CD28. (A–B) Beauvericin inhibited Akt phosphorylation in T cells stimulated with anti-CD3/anti-CD28 or Con A. Cells were harvested and whole-cell extracts were analyzed by western blot analysis. Data represent mean ± SEM of three independent experiments. *P<0.05, **P<0.01 *vs.* activation control; ^##^ *P*<0.05, *vs.* sham. (C–D) Treatment with the Akt inhibitor LY294002 (1–10 µM, 24 h) induced apoptosis of T cell stimulated with Con A; whereas VO-OHpic reversed beauvericin-induced apoptosis of the activated T cells. (E–F) Treatment with the Akt inhibitor LY294002 (1–10 µM, 24 h) suppressed cytokine production in the supernatant of T cell stimulated with Con A; whereas VO-OHpic reversed the suppressive effects of beauvericin on cytokine production. Data represent mean ± SEM. A significant difference was revealed following one-way ANOVA (*P<0.05, **P<0.01; Dunnett's test)

Besliu and colleagues [23] have proposed that PI3K/Akt signaling in peripheral T lymphocytes plays an important role in systemic lupus erythematosus. We wanted to analyze the role of this signaling axis in activated T cells, thereby supporting further consideration of PI3K/Akt as a target for limiting inflammatory diseases. Bad phosphorylation inhibition indicates the potential effects of beauvericin on Akt. In this study, T cells were isolated and challenged with Con A (5 µg/mL) or anti-CD3/anti-CD28 for 30 min. The results suggested that phosphorylation of both Thr 308 and Ser 473 in Akt were increased more in activated T cells than in naive T cells, whether stimulated with a TCR signal or a mitogen signal (Fig. 7). Beauvericin also dose dependently inhibited the phosphorylation of 2 Akt residues in activated T cells. And, LY294002, an Akt inhibitor, mimicked the effects of beauvericin, which also significantly induced T cell apoptosis (Fig. 7 B).

Moreover, we assessed whether an Akt activator could prevent or alter the effects of beauvericin on activated T cells. Administration of PTEN inhibitor VO-OHpic (which has an Akt-activating effect) (3 µM) [24], [25], significantly reversed beauvericin's effects of cytokines production suppression and apoptosis induction (Fig. 7 C). These results indicate that beauvericin inhibits activated T cells via PI3K/Akt inhibition, thereby suppressing cell activation/proliferation, reducing cytokines production and resulting in cell apoptosis.

## Discussion

Crohn's disease is an important cause of gastrointestinal disease in children and adults. It occurs worldwide, but is more common in western countries [26]. Animal models of intestinal inflammation have provided useful insight into the pathogenesis of this disease [27]. In the present study, we demonstrated that a cyclic peptide, beauvericin [28], has a marked activity on TNBS-induced colitis in mice. The intraperitoneal administration of beauvericin at doses of 2–4 mg/kg significantly reduced body weight loss and increased survival in TNBS-induced colitis. Diarrhea was also noticeably alleviated in the treated groups (Fig. 1). In the clinic, Crohn's disease often involves transmural inflammation. Histological findings include small superficial ulcerations over Peyer's patches, as well as focal chronic inflammation extending to the submucosa, sometimes accompanied by non-caseating granuloma formation[29], [30]. In our experiments, beauvericin administration inhibited inflammatory damage, such as colon edema, crypt distortion, goblet cell loss, mononuclear cell infiltration, etc. (Fig. 2). These findings suggest that beauvericin may be beneficial for treating chronic inflammatory diseases.

TNBS-induced experimental colitis is typically used to mimic clinical Crohn's disease for evaluation of *in vivo* pathological process and therapeutical effects. It is a T-cell-mediated immune response to cutaneous sensitization and subsequent challenge with haptens [31].The activation of T cells against the antigen-TNBS plays a vital role in this autoimmune-related diseases. The T cells mediating intestinal inflammation appear to be CD4^+^ lymphocytes that secrete IL-2, IFN-γ and TNF-α [32], [33], [34]. In this study, we demonstrated that beauvericin administration markedly prevented the levels of T cell-related cytokines (IFN-γ and TNF-α) and the infiltration of T cells (Fig. 1 E-F and Fig. 2 D). These findings showed the potential effect of beauvericin on T cells.

Moreover, the results of colonic cytokines analysis could also reflect an inhibition of beauvericin on other cell types. The finding that beauvericin-treated mice have lower concentrations of IL-12 and IL-1β suggests inhibition of macrophages and dendritic cells. It has been reported that beauvericin is cytotoxic on immature dendritic cells, mature dendritic cells and macrophages with IC50 equal to 1.0 µM, 2.9 µM and 2.5 µM, respectively [35]. We further obtained peritoneal macrophages stimulated them with LPS to test the activity of this compound. In deed, it showed an inhibitory effect on the production of IL-1β, IL-12 and TNF-α by macrophages (Figure S1). The antibiotic activity of beauvericin has also been known for a long time [36]. Intestinal flora and macrophages are both important factors in the etiology of inflammatory bowel disease [37], [38]. Thus beauvericin might treat colitis through these sites besides suppressing effector T cells. However, in the present study we aim to introduce beauvericin as a novel drug candidate for the treatment of Crohn's disease, which is dominated by activated T cells. Thus we focused our study on the evaluation of the efficacy and mechanism of beauvericin in suppressing T cells.

It is believed that activation and proliferation of T cells contribute to the inflammatory process in Crohn's disease [39]. In our experiments, beauvericin reduced the proliferative response of T cells after activation by Con A (Fig. 3 A). Furthermore, our results demonstrate that CD69/CD25 expression, early activation markers expressed in T lymphocytes, decreased following the beauvericin treatment (Fig. 3 B–C). This result suggests that beauvericin may interfere with naïve T cell activation.

Therefore, we assessed the cytokine profiles in supernatants of isolated T cells stimulated with Con A. The suppressive efficacy of beauvericin on IL-2, IFN-γ and TNF-α was observed in T cells activated *in vitro* (Fig. 4 A). Furthermore, IFN-γ-STAT1-T-bet signaling is involved in T-cell mediated immune response and cell survival [40]. In this study, inhibited STAT1-T-bet signaling in T cells by beauvericin may be linked to the resolution of TNBS-induced colitis (Fig. 4 B and C). This result may be very useful for treating inflammatory bowel diseases.

In addition, beauvericin induced activated T cell apoptosis in a dose-dependent manner, as demonstrated by Annexin/PI staining (Fig. 6 A–B). It is well documented that intestinal T cells exhibit resistance to multiple apoptotic signals in experimental models of colitis, as well as in IBD patients [41], [42]. This broad resistance to apoptosis accords with the fact that the T cells in inflamed tissue express increased levels of Bcl-2, and, therefore, may be resistant to a range of apoptotic mechanisms involving mitochondrial activity [43], [44]. The apoptosis induced by beauvericin in T cells had the following features: collapse of mitochondrial membrane potential (Fig. 5 A–B), release of cytochrome *c* to the cytosol (Fig. 5 C), downregulation of the anti-apoptotic protein Bcl-2 and phosphorylation of the pro-apoptotic protein Bad, activation of caspase-12, 9, 3 and PARP cleavage (Fig. 6 C and D). These findings are consistent with the data presented by Lin and Jow for human non-small cell lung cancer and leukemia cells [45], [46]. Taken together, beauvericin shows multiple effects against activated T cells: suppression of cell proliferation, activation, cytokine production, as well as induction of apoptosis.

It is still unclear how beauvericin induces T cell apoptosis. Here, we report for the first time that beauvericin induces apoptosis via the downregulation of PI3K/Akt signaling. The PI3K/Akt pathway lies at the heart of signaling networks governing cell proliferation, differentiation and survival. Akt phosphorylates Bad on Ser136, which causes phosphorylated Bad to dissociate from the Bcl-2/Bcl-X complex and lose its pro-apoptotic function [47]. Akt may also activate NF-κB via IκB kinase (IKK), thus inducing transcription of pro-survival genes [48], [49]. Phosphorylated Akt is used as a biomarker and target in cancer treatment [50], [51]. Since effects of beauvericin were closely correlated with cell proliferation and apoptosis, we had a hypothesis that beauvericin might inhibit activated T cells partly through effect on Akt. Our data showed that (1) beauvericin decreased Akt phosphorylation in a dose-dependent manner (Fig 7. A–B) in T cells stimulated with either Con A or anti-CD3/anti-CD28; (2) PI3K/Akt inhibitor LY294002 significantly induced apoptosis and suppressed cytokines production in activated T cells, which was similar to beauvericin (Fig. 7 C–F); (3) the effects of beauvericin on cytokines suppression and apoptosis induction were markedly reversed by the Akt-activating compound VO-OHpic (a PTEN inhibitor) in T cells (Fig. 7 C–F). All together, our data supported a direct action of beauvericin on T cells mainly through PI3K/Akt inhibition. Moreover, Akt activation by VO-OHpic could also reduce the inhibitory effects of beauvericin on cytokines production in LPS-stimulated macrophages (Fig S1.). Indeed it seems that the effect of beauvericin in ameliorating colitis encompasses also the effects on antigen presenting cells.

**Figure 8:**
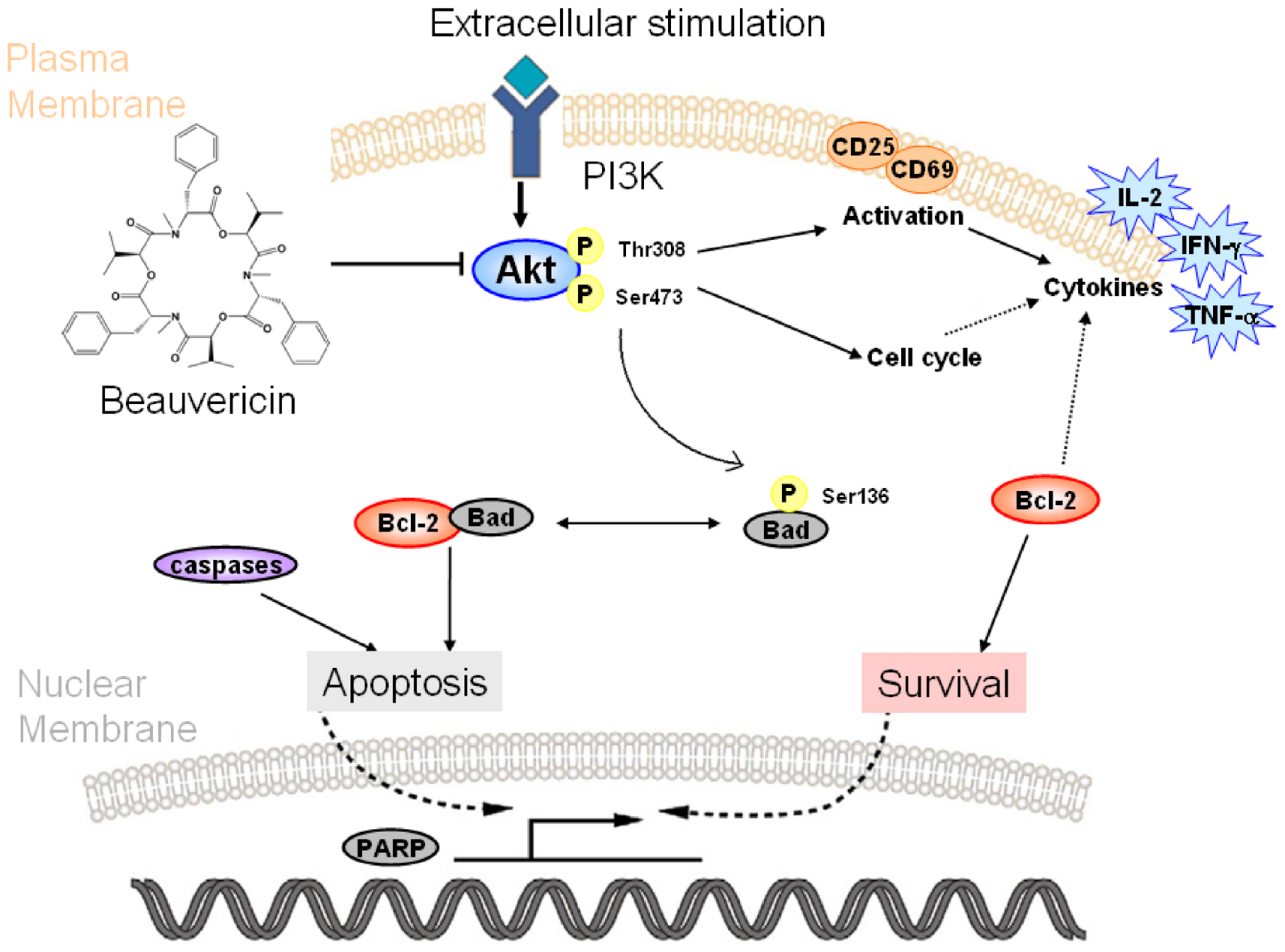
Overview of the suppressive effects of beauvericin on activated T cells targeting Akt phosphorylation. Beauvericin acts against multiple aspects of activated T cells: inhibiting proliferation and activation, suppressing cytokine profiles, and inducing apoptosis. These effects are closely associated with the downregulation of Akt phosphorylation.

Based on our findings, beauvericin has potential for use against TNBS-induced colitis. It could inhibit proliferation and activation, regulate cytokine profiles, and induce apoptosis in activated T cells. All of these effects are closely associated with its unique mechanism of suppressing PI3K/Akt signaling, which is summarized in Figure 8. In conclusion, our results indicate that targeting PI3K/Akt in activated T cells by beauvericin may yield a novel therapy for Crohn's disease.

## Supporting Information

Figure S1 **Beauvericin inhibited LPS-induced IL-1β, IL-12 and TNF-α production in macrophages.** Peritoneal macrophages elicited with thioglycollate broth were obtained from the peritoneal cavity of balb/c mice. Cells were washed twice in PBS and suspended in RPMI-1640 medium containing 10% FBS, 10,000 U/ml penicillin and 10 mg/ml streptomycin. The macrophages suspended in culture medium were cultured in 24-well microplates for 40 min at 37°C in a moist atmosphere of 5% CO_2_. Non adherent cells were removed by washing the plate twice with PBS. The adherent macrophages were used for experiments. Peritoneal macrophages were treated with various concentrations of beauvericin with/without VO-OHpic in the absence or presence of LPS (500 ng/mL) for 24 h. IL-1β (A), IL-12 (B) and TNF-α (C) in culture medium were determined by ELISA, respectively. One-way ANOVA revealed a significant difference at *P*<0.05. *P<0.05, **P<0.01 (Dunnet's test).
(TIF)
