# Supplementary figures and images for: Beauvericin Ameliorates Experimental Colitis by Inhibiting Activated T Cells via Downregulation of the PI3K/Akt Signaling Pathway
Source: PLoS One. 2013 Dec 10;8(12):e83013. doi: 10.1371/journal.pone.0083013 (PMC3858350; doi:10.1371/journal.pone.0083013)

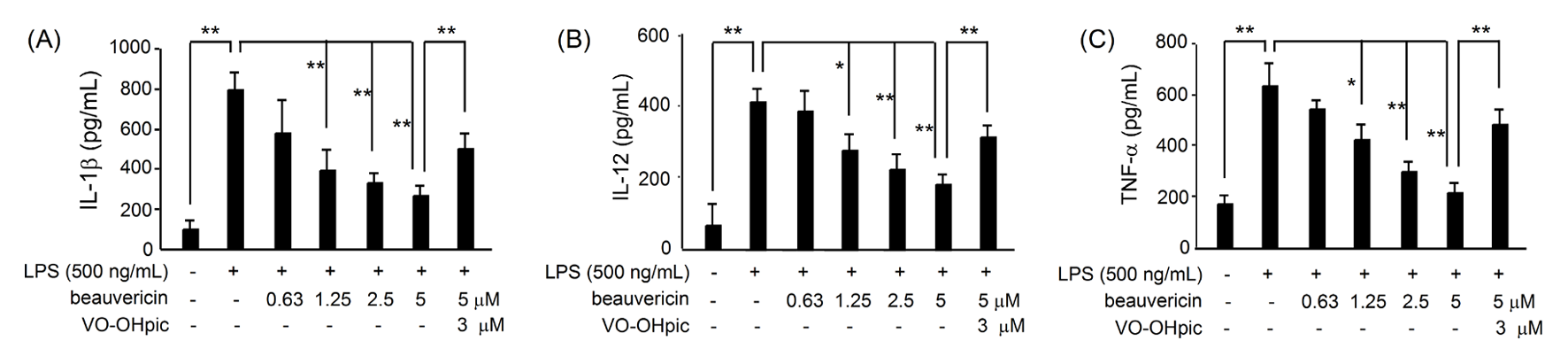

Supplement: Figure S1 — Beauvericin inhibited LPS-induced IL-1β, IL-12 and TNF-α production in macrophages. Peritoneal macrophages elicited with thioglycollate broth were obtained from the peritoneal cavity of balb/c mice. Cells were washed twice in PBS and suspended in RPMI-1640 medium containing 10% FBS, 10,000 U/ml penicillin and 10 mg/ml streptomycin. The macrophages suspended in culture medium were cultured in 24-well microplates for 40 min at 37°C in a moist atmosphere of 5% CO2. Non adherent cells were removed by washing the plate twice with PBS. The adherent macrophages were used for experiments. Peritoneal macrophages were treated with various concentrations of beauvericin with/without VO-OHpic in the absence or presence of LPS (500 ng/mL) for 24 h. IL-1β (A), IL-12 (B) and TNF-α (C) in culture medium were determined by ELISA, respectively. One-way ANOVA revealed a significant difference at P<0.05. *P<0.05, **P<0.01 (Dunnet's test). (TIF) [file pone.0083013.s001.tif]
